# Modelling and Simulation of Collaborative Surveillance for Unmanned Traffic Management

**DOI:** 10.3390/s22041498

**Published:** 2022-02-15

**Authors:** Juan A. Besada, David Carramiñana, Luca Bergesio, Ivan Campaña, Ana M. Bernardos

**Affiliations:** Information Processing and Telecommunications Center, Universidad Politécnica de Madrid, 28040 Madrid, Spain; d.carraminana@upm.es (D.C.); luca.bergesio@upm.es (L.B.); ivan.campana@upm.es (I.C.); anamaria.bernardos@upm.es (A.M.B.)

**Keywords:** unmanned traffic management, unmanned aerial system, Remote-ID, ADS-B, FLARM, drone telemetry, drone tracking, review, simulation models, collaborative, surveillance

## Abstract

Unmanned traffic management (UTM) systems rely on collaborative position reporting to track unmanned aerial system (UAS) operations over wide unsurveilled (with counter-UAS systems) areas. Many different technologies, such as Remote-ID, ADS-B, FLARM, or MLAT might be used for this purpose, in addition to the direct exploitation of C2 telemetry, relayed though cellular networks. This paper provides an overview of the most used collaborative sensors and surveillance systems in this context, analyzing their main technical parameters and performance effects. In addition, this paper proposes an abstracted general statistical simulation model covering message encoding, network capacity and access, sensors coverage and distribution, message transmission and decoding. Making use of this abstracted model, this paper proposes a particularized set of simulation models for ADS-B, FLARM and Remote-Id; it is thus useful to test their potential integration in UTM systems. Finally, a comparative analysis, based on simulation, of these systems, is performed. It is shown that the most relevant effects are those related with quantification and the potential saturation of the communication channels leading to collisions and delays.

## 1. Introduction

Unmanned aerial vehicles (UAVs), also known as drones, are becoming increasingly widespread in our societies due to their affordability, ease of use and the competitive advantage they provide for some applications [1]. For instance, drone usage is thriving for infrastructure inspections (such as for railways or power lines [2]), precision agriculture [3] or for emergency management [4]. Other applications, named urban air mobility [5], including air parcel delivery or passenger mobility, are incipient and will flourish in the medium term. As a result, the European commercial drone fleet is expected to grow rapidly according to the European Drones Outlook Study [6]. This study estimated that 400,000 commercial vehicles and 7 million recreational hulls will be operational in Europe by 2050. Consequently, unmanned traffic is expected to become prevalent in the low-level and very low-level airspaces. However, their expansion creates safety concerns including in-air incidents with manned aviation or flights over unauthorized areas (e.g., people gatherings, sensitive locations).

In this context, unmanned traffic management (UTM) systems arise as one of the main enablers for drone usage expansion while guaranteeing the safety of the rest of the aircraft and citizens [7]. These systems will allow for the efficient, concurrent, and safe operation of a high number of UAVs and manned aircraft over the same airspace. Formally, a UTM system is a set of digitized and highly automated services working collaboratively to ensure that goal. Those services allow the interaction of multiple actors (i.e., drones, pilots, operators, authorities, etc.) to enable drone operations. Two groups of interactions can be identified:

- Pre-flight interactions, consisting mainly of the authorization process that guarantees operational safety ex ante. This process is initiated at the request of the operator by sending the description of an operation or flight plan to the UTM system. The latter verifies that regulation and airspace requirements are met and that there are no conflicts with other operations (i.e., strategic conflicts between operations). If all conditions are met, the operation is authorized and registered in the UTM system to avoid later simultaneous operations in that area.
- In-flight interactions, which aim to assure the safety of ongoing operations. To do so, UTM systems enable tracking drones’ trajectories and detect conflicting aircraft in real time, using positioning reports from various sources. Meanwhile, conformance with the issued authorization and safety rules of all operations is checked, generating real-time alerts: conformance deviation; tactical conflicts between aircraft. Additionally, information about the hazardous states of UAS (i.e., loss of control, communications, navigation, etc.) needs to be rapidly passed on to both authorities and to surrounding traffic, potentially impacted by the possibly erratic behavior of the drones in these situations.

This paper focuses on the later type of (tactical) interactions, which are key to managing contingency scenarios that might occur unexpectedly. For instance, a strategically safe operation might incur in unsafe situations inflight due to a deviation from the expected trajectory (caused by pilot negligence) or the existence of rogue drones in its surroundings. Likewise, airport tower controllers or critical infrastructure administrators want to closely monitor nearby traffic to assess if and how they may affect manned aircraft operations, to act accordingly in case of unauthorized behaviors. To fulfill these workflows, UTM systems rely on the following sources of information [8]:

- Telemetry. This consists of periodic position reports sent by drones to the ground control system (GCS), typically relayed to cloud-based UTM services over public communication networks. This source of information is similar to that provided by networked remote identification.
- ATM surveillance is an information interchange used to obtain manned aircraft location. This information is typically derived from the integration of measures from a network of secondary surveillance radars (SSR, recently of the Mode S type), primary surveillance radars (PSR), wide-area multilateration (WAM) or multilaterarion (MLAT) systems, and automatic dependent surveillance (ADS-B) stations [9]. These sensors will not be covered in this paper, except if they are also used for drone surveillance.
- UAS non-cooperative surveillance networks (NCS), which may detect drones performing operations that have not been authorized and declared with the UTM ecosystem (non-cooperative drones). This information is key in some use cases, such as detecting unauthorized flights over critical infrastructures or real-time tactical conflict detection with cooperative drones or with manned aircraft. The prototypical example of this type of sensor is radar systems, but visual, acoustical or RF techniques are also used.
- UAS cooperative surveillance networks (CS). UAS positions are derived from messages sent by the aircraft themselves through a broadcast network. Contrary to the NCSs, cooperative sensors require the presence of compatible equipment on the aircraft (cooperative drones) that, being active, periodically and automatically transmits their kinematic states and some additional state information. Examples of these types of sensors include ADS-B, FLARM or direct remote identification.

All these information sources are fused within UTM tracking services to provide “consecutive surveillance observations of the same UAS flight with tracks, including the current position, heading and speed” [10]. These tracks are then forwarded to interested actors via traffic information services, which can also use network identification services to discover the identity of collaborative in-flight UAS [11]. Overall, these information sources and UTM services ensure real-time situational awareness for all actors.

At first glance, it could be argued that since NCS is needed for contingency and security purposes, CS is redundant and unnecessary. However, CS offers several advantages in terms of cost-efficiency, as a single sensor/technology can cover much greater distances with much enhanced detection and accuracy performance. It also facilitates functionalities such as aircraft identification, and subsequently conformance monitoring. In fact, both technologies complement each other, and a hybrid deployment allows increasing security by deploying NCS in critical locations (potential targets of unauthorized flights) while using CS in the rest of the areas, reducing the surveillance costs. Indeed, the coexistence of both surveillance networks is not novel. As previously described, the concurrent usage of primary (non-cooperative) and secondary (cooperative) radars is common in traditional ATM use cases.

As drone usage increases, so does the need for deploying surveillance networks that support tactical UTM functions by feeding positioning information in real time. To ensure their effectiveness, deployment planning tools are needed so that coverage in the target area is achieved while keeping costs low. In addition, it is also required to analyze their integration within UTM systems and how they affect their performance. However, no tool focused on UAS surveillance performance analysis was found in the authors’ review. A first step to develop these tools is to model the performance and behavior of each sensor (technology) so that different alternative deployment scenarios can be tested using simulations.

The main objective of the present article is to identify a set of models allowing the simulation of surveillance networks. NCS and CS underlying technologies are radically different: physical (RF, radar, acoustic, …) signals observation/detection vs. networked communication. This article focuses on modelling CS, based on a state-of-the-art review on collaborative surveillance technologies for UAS summarized in Section 2. From it, a general model to simulate CS behavior and performance (focusing on the impact on UTM systems) is proposed in Section 3. This general model is used in Section 4 to define specific simulation models tailored for the surveillance systems found in the literature review. Finally, Section 5 provides some simulation results in a collection of realistic scenarios. This is done by using and extending the agent-based UTM evaluation simulator previously proposed by the authors in [12].

## 2. State of the Art on Cooperative UAS Surveillance

Cooperative UAS surveillance technology is still under development and standardization. Initially, due to the quick emergence of drones and the lack of purpose-built technologies, traditional aviation surveillance protocols such as ADS-B (discussed in Section 2.1) or FLARM (covered in Section 2.2) have been adopted (and in the latter case adapted) for UAS surveillance. However, some authors argue that this approach may not be suitable as a long-term solution due to the limited available bandwidth and the high drone density expected in the coming decades [13].

In parallel, real-time telemetry transmission to the UTM system (described in Section 2.3) via public communication networks has become the de facto approach to provide information on cooperative drones. This approach per se is not complete for federated UTM deployment (multiple UTM services providers working jointly over a same airspace [14]) projected in many countries. Federated deployment is favored due to its expected economic advantages due to competitiveness. On the other hand, the lack of common protocols and data models among UTM providers would oblige drone operators and manufactures to adapt their aircraft/GCSs for each service provider. Additionally, gateways among them would be necessary to provide full situational awareness to all actors and therefore guarantee safety. 

To simplify this problem, a standardized and purpose-crafted solution for UAS collaborative surveillance is needed. This standardization effort is still ongoing, with similar regulations in the United States [15] and Europe [16] requiring the “Remote Identification” of drones, defined as “the ability of a drone in flight to provide identification and location information that can be received by other parties” [17]. The requirements defined in these regulations are the base for the development of some existing technical standards such as ASTM F3411—19 or AD-STAN prEN 4709-002 that will be discussed later in Section 2.4.

Although the previous legal requirements and technical standards set the path for the near future, discussion on how to implement cooperative UAS surveillance is still ongoing, as will be shown in Section 2.5. First, a discussion exists as to whether broadcast-based alternatives (position reports broadcasted through a physical channel in an area around the drone) would be preferred over networked approaches (the drone directly sends position reports to the UTM system over the internet). Second, privacy concerns with regards to how a drone must be identified or who shall have access to that surveillance information are still unresolved. Next, we will describe some of the most prevalent solutions. 

### 2.1. ADS-B

ADS-B [18] is the acronym of Automatic Dependent Surveillance-Broadcast. It is a surveillance technology based on the periodic broadcast of on-board navigation (typically GNSS) technologies position estimation. The system is “Automatic” because it does not require any interrogation signals from the ground, and it is “Dependent” because the quality of the position detected depends on the sensors installed on the aircraft. Since the signal is broadcasted, it can also be received by nearby aircrafts to provide situational awareness and allow self-separation, without the intervention of other entities. Apart from the benefits directly derived from its technical features (specifically, high accuracy and integrity), one of the other main benefits is the reduced cost of the system: ground stations are significantly cheaper than primary and secondary radar systems used by air traffic control.

The system may work on two different carrier frequencies: 1090 MHz, which is the default frequency, and 978 MHz, preferred for flying altitudes below 18,000 feet with the aim of reducing the congestion on 1090 MHz at the lowest altitudes.

ADS-B relies on two avionics components aboard each aircraft: a high-integrity satellite navigation source (i.e., a certified GNSS receiver) and a datalink (the ADS-B unit). The ADS-B unit defines the equipage class category of the aircraft or of the ground system. Class A units provide the interactive capability among aircrafts, Class B units only broadcast the information, and Class C units are reserved for the ground receiver systems. Classes A and B are divided into four subclasses depending on their antenna: Classes A0 and B0 have the lowest transmission power and the lowest sensitive antennas, while A4 and B4 have the highest transmission power and the most sensitive receivers. Depending on the antenna diversity, each subclass can also provide a different set of features in addition to the basic ones. Class C has three subclasses, named C1 to C3, each of them with different sensitive receivers and different surveillance capabilities.

The typical information provided by the ADS-B is the identification of the aircraft, the current position (longitude and latitude), altitude and velocity. Other information may be transmitted and are described in the standard, such as weather or flight information. Messages (called “squitters”) are typically broadcasted once every 2 s, but the data contained in the packets are updated at different rates depending on the information (e.g., the position is updated at most every 0.2 s, the velocity at most every 1.3 s, etc.) and the broadcast frequency can be increased up to two messages per second. The packets have a size of 56 bits or 112 bits. Short squitters are packets of 56 bits which only contain 8 bits of packet information, 24 bits of aircraft identification and 24 bits of parity. Long squitters (112 bits) have 8 bits of packet information, 24 bits of aircraft identification, 56 bits of data and 24 bits of parity. Since the packets are only 56 or 112 bits, a lot of effort has been devoted to compressing as much information into the packets as possible. No medium access protocol is implemented, packets can interfere, but the very short messages (max 120 μs), the low frequency of broadcast permitted (max 2 Hz) and the large number of receivers reduce the probability of collision and increase the probability of a reception. ADS-B is also currently susceptible to attacks and security issues, not providing any authentication and encryption features. Data fusion and other techniques may be implemented to reduce the impact of these issues. A summary of the discussed technical characteristics of ADS-B is shown in Table 1.

Nowadays, ADS-B is incorporated in most manned aircrafts and the operators are encouraged to install ADS-B equipment. It is a good surveillance method for the flights over areas not covered by traditional radar. In the context of the UAS, ADS-B transceivers on small UAS are no longer limited by payload and power capabilities (e.g., the transceiver in [19] has a size of 50 × 25 × 17 mm and a weight of 20 g), and they represent promising opportunities for the regulated operation of small UAS at a reduced cost. However, the use of ADS-B on UAS is still under study. The lack of a medium access protocol could represent a limitation for UAS, where a huge number of aircrafts can potentially fly in a relatively reduced space. In [13,20], the authors analyze this scenario through several simulations, coming to the conclusion that it will probably be necessary to balance the demand of air space occupation to provide a good safety level to all the aircrafts. In [21], a similar scenario was studied, also considering the current regulatory practices of the U.S. National Airspace System, and the authors finally reported that ADS-B should be used along with some other system, especially optical sensors for collision avoidance. Other works were focused on the simulation of the ADS-B mounted on UAS to evaluate performance at low altitudes [22] or to propose collision-avoidance algorithms based on the ADS-B information [23].

### 2.2. FLARM

Traditionally, Flight Alarm (FLARM) was a traffic awareness and collision avoidance technology designed for the needs of light aviation (i.e., gliders, helicopters, etc.), similar to ADS-B. Based on this technology, an electronic identification system was released in 2018, known as “FLARM UAS Electronic ID” [24]. FLARM broadcasts a secure electronic identification and 3D position of a given UAS via a radio frequency digital message.

The FLARM system consists of a GNSS receiver for localization and timing, a processor to manage the protocol and a RF physical transmitter. The transmitter broadcasts the protocol messages over the unlicensed 868.4 MHz band using a digital modulation scheme. Carrier-sense multiple access is used for medium access, listening for other transmissions on the channel and deferring the transmission a random time if it has been used. Therefore, packet collisions and transmission delays might occur.

Two different messages are to be transmitted over this physical layer: UAS identification messages and operator identification messages, indicating the identity and position (longitude, latitude and MSL/WGS84 altitude) of the UAS and its operator, respectively, at a given time. By default, messages are transmitted once every 3 s. The protocol uses the manufacturer serial number as the drone identification. 

Messages consist of a preamble, a sync word, a partially encrypted payload with the message information, a CRC code for integrity and an optional message signature for UAS authentication. Using this last field, the protocol describes a public-private key mechanism to ensure transmitter (UAS) identity using a registration service for verification. Table 2 summarizes the main technical features of the protocol:

Multiple commercial products exist implementing the FLARM electronic identification protocol (often along with other technologies described also in this review). For instance, the Atom UAV [25] dongle from FLARM includes a FLARM transceiver, an ADS-B receiver and an ASTM F3411-19 emitter. Droniq HoD [26] also implements a FLARM transceiver along with ADS-B IN and telemetry transmission over LTE. Commercial ground receivers (sensors) also exist such as GBSAS stationary from Droniq [27] with up to 20 km range for FLARM and 100 km range for ADS-B.

### 2.3. Direct Telemetry Reporting

As previously stated, periodic telemetry reporting is currently the main procedure to provide real-time position information to UTM systems. It consists of using public mobile networks (i.e., 3G, LTE) to directly relay messages to the UTM system over the internet. Thus, it inherits the physical limitations (i.e., coverage, latency, speed, etc.) of the used networks. Although the physical layer in this approach is inherently standardized, no uniform protocol currently exists for defining data models or expected interactions. Thus, each UTM provider (e.g., AIRMAP, Altitude Angel, Droniq, Unifly…) defines its own data models through well-defined API that are distributed to drone manufacturers and developers. As a result, this approach requires adaptations in the connected device (usually an on-board dongle or the GCS) for each provider.

AIRMAP provides a mobile SDK for iOS and Android to abstract developers and manufacturers from the internal transmission protocol. The idea is to develop a companion GCS app (for instance, one developed using DJI Mobile SDK for DJI drones) that is integrated into the AIRMAP UTM ecosystem using its own SDK. A series of utilities are then available within the SDK [28] to easily publish telemetry data (position, attitude and speed) to the UTM system. Internally, the SDK translates the high-level API into a series of remote procedure calls (using gRPC) that transmit the telemetry information encoded into protobuf, (a serialization protocol for structured data) messages [29]. 

Similarly, Altitude Angel also provides a well-defined API to send telemetry information to the UTM system [30]. In this case, a REST interface is used to send JSON messages. Each position report includes a timestamp, the UAS position, altitude, ground and air velocity, accelerations and heading. In addition, Altitude Angel has also defined an open-source hardware and software architecture aimed at developing drone-attachable dongles that transmit a drone’s position. The architecture, named Scout [31], is based on on-the-shelf components and can be used by any drone developer to integrate a drone into any UTM system (although the provided code is developed for the Altitude Angel API).

Finally, other UTM service providers have developed custom, proprietary, on-board dongles to send telemetry information from the drone via LTE. That is the case of the aforementioned Dronic HoD [26] or Unifly BLIP [32].

### 2.4. Remote Identification

European drone regulations ruled in 2019 that all UAS over 250 g must have the means to provide direct remote identification functionality (unless physically tethered). In particular, drones must be able to perform “real time […] direct periodic broadcast using an open and documented transmission protocol […] in a way that can be received directly by existing mobile devices within the broadcasting range” [16]. This broadcast consists of a unique (manufacturer-provided) UAS serial number, a UAS operator registration number, the geographical position of the drone with its height above the take-off point, the course and speed of the drone, its emergency status and the geographical position of the operator. A later amendment of the said European regulation [33] also introduced optional network remote identification, transmitting the same information as in the direct (broadcast) remote identification. Identification information is expected to be processed in a “network identification service” in UTM systems [11]. Thus, authorized users (including general population in the flight area and competent authorities) must be able to access the remote identification information of in-flight drones.

A similar regulation enacting the need for broadcasting remote identification has recently been endorsed in the United States [15]. UAS must broadcast (themselves or using a remote identification broadcast module) the following information: UAS identification, the latitude/longitude, altitude, and velocity of the UAS, the latitude/longitude and altitude of the control station, emergency status and a time mark. This broadcast must be performed using a protocol compatible with existing personal wireless devices. To ensure UAS operation privacy, the UAS identification can use a temporal session id, which can only be correlated with the registration database by authorities. Contrary to European regulations, network-based remote identification is not considered. In addition, the rule also prohibits the use of ADS-B as a means of meeting remote identification requirements. Regulators argue that its usage shall be limited (prior authorization) when large UAS are operating in controlled airspace.

The foregoing regulations prompt the need to develop open and standardized broadcast transmission protocols that can be received using mobile devices. This limits the possible physical layers to mainly Wi-Fi and Bluetooth (although protocols such as LoRa have also been explored [34]). Currently, one RemoteID protocol is already established mainly inspired on US regulations: ASTM F3411-19 [35]. In parallel, at least two other standards are under development. On one hand, ASD-STAN is developing the prEN 4709-002 standard inspired by European regulations, seeking compatibility with the ASTM standard [36]. On the other, the IETF drip group [37] is also developing a RemoteID protocol leveraging existing internet protocols.

Focusing on the ASTM standard, the broadcasting of identification messages is performed using existing broadcast frames (usually used as advertisements to establish connections) within the Bluetooth (legacy or 5.x LE) and Wi-Fi protocols. The idea is to use the payload of these frames to transmit a short ad hoc id message, thus eliminating the need to extend physical protocols. Two main compulsory message types are considered: a static one broadcasted every 3 s with drone identity, and a dynamic one broadcasted at least each second with UAS location. Additionally, the protocol also provides a set of optional messages that can be used for operator identification, operator location transmission, transmitter authentication. Table 3 summarizes the main technical specifications of the ASTM standard.

The ASTM protocol also standardizes a protocol for the networked identification of drones using an adaptation of the previous messages to internet friendly formats. To do so, it decouples the network identification service into two different roles: service providers and display providers. Service providers implement networked communications with drones receiving and processing their message (even providing extrapolated information). Display providers serve as a gateway to end users supplying them with the traffic information in their area of interest. This decomposition paves the way for federated UTM systems. In fact, the protocol also defines a discovery and synchronization protocol for USS interoperability that is outside of the scope of this paper.

It is expected that new drones directly implement their own means to fulfill RemoteID requirements (e.g.,: DJI statement in [40]). For instance, some DJI [41] and Parrot [42] drones already support some remote identification capabilities via software update and repurposing the C2 channel. Meanwhile, dongles or broadcast modules are to be used for this purpose. Some commercial examples include Aerobits idME+ [43] or ScaleFlyt [44] from Thales, both compatible with ASTM standard. Sensors/receivers for RemoteID seem to still be under development. In particular, no smartphone-based receiver has been found. Some companies’ commercial announcements publicize RemoteID receivers (e.g.,: [45,46]), but with no technical details that allow us to assess their technological maturity.

### 2.5. Ongoing Discussion

The main open discussion analyzes the usage of networked or direct/broadcast remote identification. First, networked solutions’ dependence on public mobile communication networks limits the possible coverage of this solution, especially in rural areas that usually lack ubiquitous and reliable mobile connectivity. In fact, if networked identification is deemed compulsory, this would prevent developing operations in rural areas or in emergency scenarios where communications are degraded. However, proponents of networked solutions argue that a broadcast-only solution can lead to frequency congestion in unlicensed spectrum as the number of drones increases in the future. Furthermore, cooperative surveillance is expected to be used not just for traffic management purposes, but also for tactical drone safety using detect and avoid (DAA) techniques. In this case, broadcast-based alternatives would be preferred over networked approaches to avoid round-trip transmission delays. For the time being, regulations only mandate the usage of broadcast identification, while the networked approach remains optional. 

Apart from the summarized discussion on the technical aptitude of each solution, additional points regarding safety and privacy have also been discussed. For instance, networked solutions can be exposed to DDoS attacks by nefarious actors, which can jeopardize the safety of the system. Additionally, there is no consensus on who should be able to access remote identification information. Some drone operators are reluctant to send their identity and critical mission information to third party UTM systems, arguing privacy, business, and tactical issues. In this regard, discussion is still ongoing as to whether some identification positions must be encrypted and only available to authorities. Privacy is also considered when discussing if a unique drone id (as seems to be the case in the European approach) or a session ID must be used. The latter approach would allow enhanced privacy for users, but authorities must still be able to correlate that temporal ID with drone and operator real identities for accountability purposes. A good summary of the different points of view of the industry regarding collaborative surveillance (and particularly remote id) can be found in the public comments sections of the FAA final rule on RemoteID [15]. As the discussion continues, so must the standardization process (although solutions might be transient) to start enabling safe drone operations.

## 3. Collaborative Surveillance System General Modelling

The previous section has covered the main technical features of some of the existing collaborative surveillance systems for UAS. The objective of this section is to define and model the main factors that determine the performance of such systems and the overall surveillance deployment integrated within a UTM system. The aim is not to reliably reproduce the internal functioning of those systems, but to provide a statistical model that allows one to simulate their outputs. To do so, we follow a top-down approach defining a general abstracted model that can be adapted to each of the existing surveillance protocols.

Apart from telemetry reporting (already modelled by the authors in [12], and not to be covered in this paper), all systems consist of two different elements: an onboard transmitter and a ground-located receiver or sensor. Regarding the transmitter, it broadcasts GNSS positioning information of the drone over a physical channel. GNSS positioning is not free from suffering errors due to multipath propagation, clock errors, atmospheric propagation disturbances, etc. A simple model to simulate such errors was proposed by authors in [12], and will be used here to generate the input information used by each transmitter. Once a GNSS measurement is obtained, each surveillance system encodes it using different resolution and message structures. Then, two types of messages can be generated, either periodically or due to protocol-defined events such as important position changes. These messages may contain only UAS identification information, or identification plus encoded positioning/kinematic information. These messages are finally broadcasted over a RF physical layer for which a MAC protocol is required.

As a signal is propagated and received, it suffers attenuation, interferences, presence of noise, etc., which may result in digital information degradation (bit errors, burst errors…) at the receiver (sensor). Within each sensor, the physical layer characteristics and environmental conditions determine the receiving range and quality. Concurrent access to the shared RF medium can also affect the receiving capacity of the receptor and should also be modelled. Even if the digital signal has been successfully detected by the receiver, it does not guarantee that corresponding message can be decoded. This will depend on the number of transmission-generated errors (which depends on signal quality, interferences, etc.) and the capacity of the protocol to correct such errors. Finally, the receiver will process the received information and generate position reports (including all drones and information from multiple messages) that will be sent to a UTM system over public communication networks. The coverage and performance of these networks also affect the capacity of a collaborative sensor to reliably provide a UTM system with information. However, the effect of the connection from sensors to the UTM will not be considered here, as the authors covered this specific topic in a previous paper [12]. 

**Figure 1:**
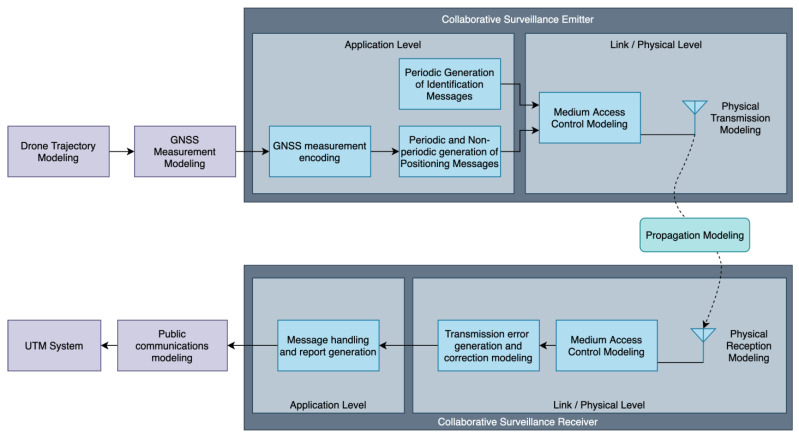
Proposed model for UAS collaborative surveillance. Blocks in purple are outside of the scope of this paper as they have already been covered by the authors in [12].

Therefore, we can identify the main elements to model collaborative systems. The aggregation of all these elements in Figure 1 constitutes the proposed simulation model:

- Collaborative surveillance emitter.
  ○ GNSS measurement encoding, which determines which information is sent (e.g., FLARM does not include speed information whereas RemoteID does) and the resolution of each element within a measure.
  ○ Message generation frequency and other conditions that trigger messages, which determines the number of messages of each type to be generated and their timing.
  ○ Medium access control. Depending on each of the protocols, emitters may be required to sense the RF medium before transmitting to avoid collisions. This can yield to transmission delays and depends on the number of emitters within the area.
  ○ Physical transmission. The emitter RF module propagates a signal with the message in a specific frequency, from the drone location and with a given transmission power and directionality.
- RF propagation. Depending on the propagation environment (urban/rural) and frequency, different attenuations and noise levels are expected.
- Collaborative surveillance receiver.
  ○ Physical receiving. The receiver requires a minimum signal level to detect the emitter message which determines the receiving range.
  ○ Medium access control. Due to the possibility of collisions when concurrently using the RF medium and depending on the MAC protocol, packets may be discarded by the receiver.
  ○ Transmission error generation and correction. The transmission errors are to be simulated within this module considering the digital signal quality. Depending on the capacity of the protocol to correct such errors, messages may also be discarded here by the receiver.
  ○ Message handling and report generation. The receiver may perform additional data processing with the incoming messages such as information filtering and prediction. The frequency for which aggregated reports are generated is also to be considered.

Next, we propose a general model for each of the previous elements. These general models will be common for all surveillance protocols, but some parameters will be dependent on the technical characteristics of each protocol. This particularization of the general models is covered in Section 4.

### 3.1. GNSS Measurement Encoding

To model the information forwarded by each protocol and the resolution used to forward that information, a list of the information elements included by each protocol can be defined as follows:

(1) $$P^{protocol}\subseteq P$$

(2) P={timestamp, longitude, latitude, altitude, speed}

where *P* is the set of all possible state information variables provided by the drone or GNSS system and protocol denotes the surveillance system the list refers to. Then, for each element of the said list or subset, a minimum resolution ($r_{p}^{protocol}$) must be defined, understood as the minimum change in the value representable with the protocol message encoding:

(3) $$r_{p}^{protocol}\;,\;\forall \;p\;\in P^{protocol}$$

Finally, it is possible to encode the information forwarded by a given protocol with the required resolution as follows:

(4) $$m_{p}^{encoded}=\lceil \frac{m_{p}^{drone}}{r_{p}^{protocol}}\rceil \cdot r_{p}^{protocol}\;,\;\forall \;p\;\in P^{protocol}\;$$

where ⌈·⌉ represents rounding to the nearest integer and $m_{p}^{drone}$ refers to the measured variable or state in its original precision. This encoding is only performed for the state variables forwarded by the protocol.

### 3.2. Message Generation

As with most of the protocols reviewed in Section 2, two different types of messages are considered in our model:

- Position report message. This message is generated with a $f_{position}^{protocol}\;\left[Hz\right]$ frequency and includes the encoded position information $m_{p}^{encoded}=\forall \;p\;\in P^{protocol}$ and drone identification information.
- Identification message. This message is generated with a $f_{identification}^{protocol}\;\left[Hz\right]$ frequency and only includes drone identification information. As it is not yet clear which information is to be used in this case, our implementation of the model uses the serial number of each drone for identification purposes. In addition, this message may be omitted in the implementation of each protocol model, as it is not considered in some cases.

For simplicity in the foregoing modelling, it is assumed that the payload of both messages is of equal length: $L_{payload}^{protocol}\;\left[B\right]$ and that each message has an overhead of $L_{overhead}^{protocol}\;\left[B\right]$ (to include both MAC and PHY levels frames overhead), for a total length of $L_{payload}^{protocol}+L_{overhead}^{protocol}\;\left[B\right]$. In fact, this simplification is accurate in most of the reviewed protocols as they used a fixed-length frame. In addition, as the processed information of each receiver is to be forwarded to a UTM system over the Internet using Internet-based formats, it is not required to simulate the binary encoding of the messages. Therefore, our implementation of this model in our simulator directly uses JSON data formats but respects the encoding resolution.

**Figure 2:**
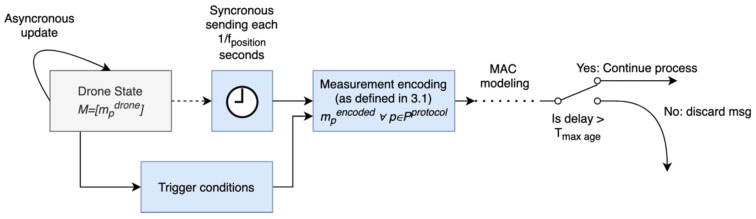
Position message generation process. The figure is a block diagram representing the different processes and information flow within the model. In general, each block represents a process or function within the model with arrows representing dependencies or the information flow. Additionally, the clock block represents a synchronous/timed process, and switches represent a decision which depends on the stated condition or probabilistic term.

Regarding the position message generation process, it can be modelled as two decoupled parallel processes, as seen in Figure 2. On one hand, the drone or the GNSS module asynchronously provides the drone state (which can be represented as a state vector composed of the measurements used in the previous step: $M=\left[m_{p}^{drone}\right],\;\forall \;p\in P$) to the surveillance emitter. This information can be provided periodically or when drone position changes, depending on the drone or GNSS module specifics. On the other hand, the surveillance emitter periodically encodes and sends the last available information every $1/f_{position}$ seconds. Due to this decoupling and some additional delays in the sending process that will be discussed later, the information sent by the emitter might not be up to date. Therefore, some protocols limit the longevity of the sent information ($m_{time}^{drone}-sending\;time$) to an amount of time $T_{max\;age}^{protocol}\;\left[s\right]$. If this condition is not met, messages are discarded. Additionally, some additional conditions might be defined in each protocol to trigger a new position message sending.

### 3.3. Medium Access Control

**Figure 3:**
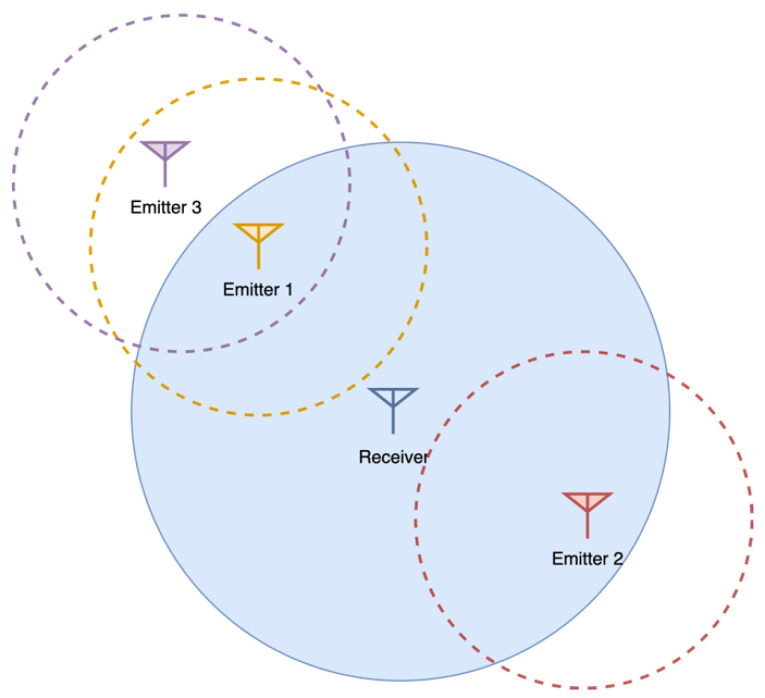
Different situations in the RF medium. Emitter 2 is a hidden node of emitter 1. Emitter 1 detects and tries to avoid messages from emitter 3. This avoidance would not be needed as emitter 3 is out of the range of the receiver.

When multiple users concurrently access a unique shared RF channel, if more than one user transmits simultaneously, information may be corrupted (in a situation known as collision) depending on the users’ geographical location and timing. Medium access control (MAC) techniques and rules are used to minimize these collisions. The simplest technique consists of listening to the channel for other transmissions before transmitting. However, even when listening for other transmissions, collisions might occur due to the propagation delay or geographical distribution of nodes. Let us imagine a situation (depicted in Figure 3) in which a principal emitter (emitter 1) wants to transmit information to a receiver in the presence of other emitters (emitter 2 and 3). In the figure, the sensing ranges (distances from which they can sense other emitters transmissions) of each node are depicted. The emitters can be classified in four different sets and subsets:

- Emitters that are within the sensing range of the main emitter, $S^{emitter}$. These are the emitters for which the main emitter may avoid collisions in some cases (depending on timing because of propagation delay), for all the possible receivers.
- Emitters that are within sensing range of the receiver, $S^{receiver}$. These are all the emitters that can cause collisions in the receiver when concurrently sending information. Two subsets of emitters can be identified here, depending on its relationship with the main emitter:
  ○ Emitters sensed by the main emitter, for which collisions may be avoided to some extent for this receiver, $S_{sensed}=S^{emitter}\;\cap \;S^{receiver}$
  ○ Emitters not sensed by the main emitter (known as hidden nodes), $S_{hidden}=S^{receiver}-S_{sensed}$, and for which collisions cannot be avoided in any case.

Some of the reviewed surveillance systems use the carrier sense multiple access (CSMA) MAC protocol [47]. The main idea behind this protocol is the previously described “listen before transmit” concept. Some variations of the protocol include a random wait before retrying a transmission (non-persistent CSMA) after the channel is sensed busy while others transmit as soon as it is free (persistent CSMA). Other surveillance protocols do not implement any explicit MAC protocol: nodes access the channel in a completely unsynchronized manner; this is called the Aloha access mechanism [48]. Therefore, the proposed MAC model must be able to adapt to this variety of MAC protocols. From this discussion, some ideas can be extracted:

- If a CSMA protocol is implemented, there is a probability that the channel is sensed as occupied, and this probability depends among other things on the traffic generated by the nodes in $S^{emitter}:$
  $$P_{busy}=f\left(S^{emitter},\;\mathrm{traffic}\;\mathrm{characteristics},\;\mathrm{distance}\;\mathrm{between}\;\mathrm{nodes}\;\mathrm{in}\;S^{emitter}\right)$$
- If a non-persistent CSMA protocol is used, a random wait in the range $\left(0,\;T_{backoff}\;\left[s\right]\;\right]$ must be used before sensing the channel again.
- When using a CSMA protocol, the collision probability in the receiver is reduced but only for those nodes included in $S_{sensed}:$
  $$P_{collision\;known\;nodes}=f\left(S_{sensed},\;\mathrm{traffic}\;\mathrm{characteristics},{\;\mathrm{distance}\;\mathrm{between}\;\mathrm{nodes}\;\mathrm{in}\;S}_{sensed}\right)$$
- In any case, unknown known nodes (to the emitter) may exist (all nodes if using Aloha), which will lead to collisions with a higher collision probability:
  $$P_{collision\;hidden\;nodes}=f\left(S_{hidden},\;\mathrm{traffic}\;\mathrm{characteristics},\;\mathrm{distance}\;\mathrm{between}\;\mathrm{nodes}\;\mathrm{in}\;S_{hidden}\right)$$

**Figure 4:**
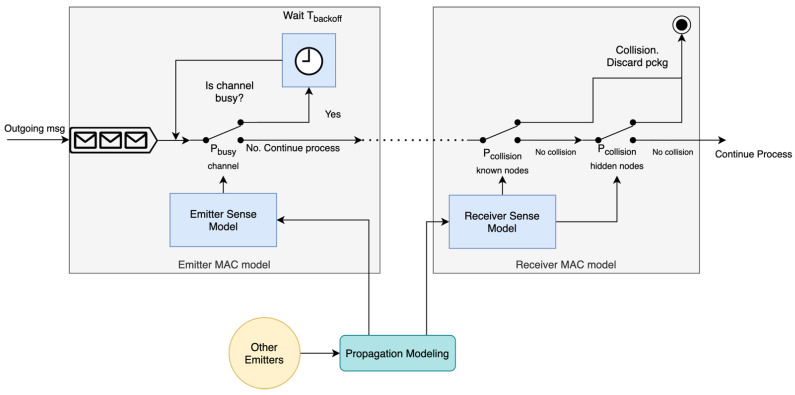
MAC model for an emitter and receiver. The figure is a block diagram representing the different processes and information flow within the model. In general, each block represents a process or function within the model with arrows representing dependencies or the information flow. Additionally, the clock block represents a synchronous/timed process, and switches represent a decision which depends on the stated condition or probabilistic term. The initial block in the figure represents a message queue for FIFO processing.

These ideas are reflected in the model MAC effect model depicted in Figure 4. First, channel usage is simulated using $P_{busy}$. If the channel is busy, a random wait can be simulated before sensing again the channel (i.e., generating a new binary decision). Otherwise, the simulation process continues. Messages are handled one by one in arriving order, meaning that if a package is waiting for a free channel, the following package waits in a queue. On the receiver end, collisions are simulated using $P_{collision\;known\;nodes}$ and $P_{collision\;hidden\;nodes}$ (by generating random binary decisions with these probabilities). It is assumed that a collision completely invalidates the message (this may not be true in real systems due to possible decoding of overlapping messages in some cases), and it is therefore discarded. 

In our initial models, we assume that each UAS surveillance system utilizes an independent communication channel. This means not only that traffic from two different systems does not interact with each other (even though some systems share bandwidth), but also that no other external users use that bandwidth. This approximation greatly simplifies the analysis but might reduce the validity of RemoteID and FLARM models, using ISM bands in which other traffic (e.g., BT, WiFi…) exists.

The probabilities defined in the model will be specified for each of the surveillance systems depending on the MAC protocol they use. The emitters of each of the defined sets can be dynamically computed in simulation time using the emitters and receivers’ position and the physical transmission model defined in Section 3.4. However, some common notations and assumptions (based on [49]) can be introduced to define the traffic generated in a given channel. It is assumed that the traffic generated by all surveillance emitters can be modelled as an independent Poisson source (this hypothesis is valid for a large number of drones, as no sending synchronization is expected). As previously stated, we also assume that all packets are of a constant length and that discarded packages are not retransmitted. Under these conditions, we can define an aggregate traffic source for the nodes in each set of nodes S. This source will have a mean packet generation rate of:

(5) $$\lambda_{S}^{protocol}\;\left[\frac{pckt}{s}\right]=N_{emitters\;in\;S}\cdot \left(f_{position}^{protocol}+f_{identification}^{protocol}\right)$$

Finally, the normalized traffic offered to the network, *G*, generated by this source can be computed as follows. This traffic that the sources generate, is not that which will be finally carried over the network. The real throughput will be lower due to the collision effect.

(6) $$G_{S}^{protocol}=\lambda_{S}^{protocol}\cdot T_{pckt}^{protocol}$$

where $T_{pckt}^{protocol}$ [*s*] is the packet transmission time which depends on the channel physical capacity or physical bit rate, $R^{protocol}\;\left[bps\right]$ (this bitrate is the gross bitrate measured at the physical layer, not the achievable throughput at the application level considering protocol overhead and encoding redundancy), and can be computed as:

(7) $$T_{pckt}^{protocol}\left[s\right]=\frac{8\cdot (L_{payload}^{protocol}+L_{overhead}^{protocol})}{R^{protocol}\cdot {\mathrm{FEC}}^{protocol}}$$

where ${\mathrm{FEC}}^{protocol}$ is the coding rate used by the protocol in those cases with Forward error correction (FEC). That is, ${\mathrm{FEC}}^{protocol}$ is the ratio of actual information over the total bits transmitted due to the additional redundancy included.

### 3.4. Physical Transmission, Propagation and Reception

Although complex physical transmission models could be proposed, this detailed approach is outside of the scope of this paper, where we are interested in the systematic effects to the UTM messages. Therefore, we propose a go-no go model based on the power balance of the radio link assuming free space losses and omnidirectional antennas. Thus, a transmission power is defined for each emitter depending on the protocol: $P_{tx}^{protocol}\;\left[dBm\right]$. Likewise, the transmission frequency will be defined for each system as $f_{tx}^{protocol}\;\left[GHz\right]$. Each network node also requires an associated position (node position in the case of the emitter and surveillance sensor position for the receiver) so that the distance between each emitter and receiver can be computed: $d_{e-r}\;\left[km\right]$. With this information, it is possible to compute the power in the receiver as [50]:

(8) $$P_{rx}\;\left[dBm\right]=P_{tx}^{protocol}-20\log d_{e-r}-20\log f_{tx}^{protocol}-92.45$$

**Figure 5:**
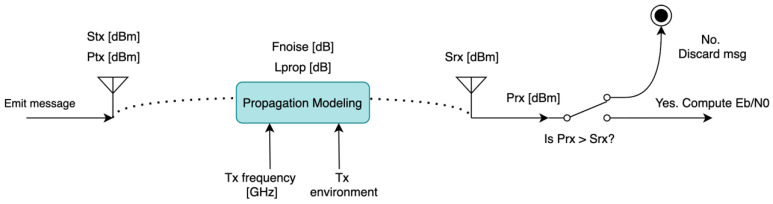
Physical transmission model. The figure is a block diagram representing the different processes and information flow within the model. In general, each block represents a process or function within the model with arrows representing dependencies or the information flow. Additionally, switches represent a decision which depends on the stated condition or probabilistic term.

To decide if a message is received, it can be compared with the receiver sensitivity $S_{rx}\;\left[dBm\right]$, defined as the minimum power required to detect an incoming signal. Likewise, emitters also have an associated sensitivity, $S_{tx}\;\left[dBm\right]$ in those cases where a “listen and transmit” (such as CSMA) procedure is used. Therefore, for a signal to be received, it must fulfill:

(9) $$P_{rx}\geq S$$

where *S* is either the emitter or receiver sensitivity as appropriate. However, attenuation is not the only effect suffered by the signal. The antenna receives the signal with an aggregated level of noise which degrades the signal. We do not consider the effect of other possible interferences. Assuming an additive white Gaussian noise (AWGN) channel, the signal quality can be estimated by the $\frac{E_{b}}{N_{0}}$ metric, which is a normalized version of the SNR for digital signals. The former can be computed as:

(10) $$\frac{E_{b}}{N_{0}}=\frac{P_{rx}\;\left[W\right]/R_{protocol}}{N_{0}\;\left[\frac{W}{Hz}\right]}$$

where $N_{0}$ is the noise power spectral density and can be computed from the overall noise factor of the system, $F_{eq}^{protocol}\;\left[dB\right]$:

(11) $$N_{0}=k\cdot T_{0}\cdot 10^{\frac{F_{eq}^{protocol}}{10}}$$

where *k* is the Boltzmann constant ($1.38\times 10^{-23}\;J/K$) and $T_{0}=290\;K$ is the reference temperature. The overall noise factor of the system is dependent on the transmission frequency (different noise sources are predominant for different frequencies) and the receiver quality [51]. The proposed model is summarized in Figure 5:

### 3.5. Transmission Errors and Error Correction

Additive noise in a digital signal manifests as a set of bit errors that might be recovered with error correction techniques or that yield the package undecodable. Burst errors might also appear due to interferences or multipath effects. In our model, we consider the effect of isolated bit errors. For a given modulation scheme, it is possible to derive theoretical expressions relating the $\frac{E_{b}}{N_{0}}$ metric to the mean number of expected errors, or bit error rate (BER). Thus, it exists a function, named $b^{protocol}$, such as:

(12) $$\mathrm{BER}=b^{protocol}\left(\frac{E_{b}}{N_{0}}\right)$$

Once this mean error rate is computed, it is possible to statistically simulate the number of errors in a message. To do so, we consider a Bernouilli experiment with BER probability repeated for each bit of the message. Overall, the number of errors in the message can be obtained with a binomial distribution:

(13) $$N_{errors}=ℬ\left(L_{payload}^{protocol}+L_{overhead}^{protocol},\;\mathrm{BER}\right)$$

**Figure 6:**
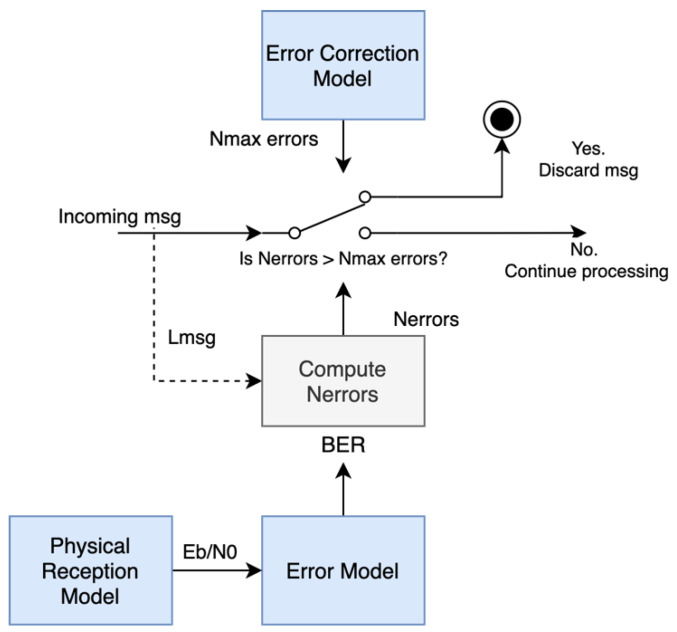
Transmission errors and error correction model. The figure is a block diagram representing the different processes and information flow within the model. In general, each block represents a process or function within the model with arrows representing dependencies or the information flow. Additionally, switches represent a decision which depends on the stated condition or probabilistic term.

As all protocols include an error detection algorithm, we assume that the probability of undetected errors is negligible. Therefore, packages with errors (or for which errors cannot be corrected) will be discarded and will not result in corrupted decoding of data. Although some error correction algorithms such as convolutional FEC correct errors within a bitstream and not in an isolated manner, we model the error correcting capacity of a given protocol as a maximum number of correctable errors: $N_{max\;errors}^{protocol}$. Therefore, a message is decodable if $N_{errors}<N_{max\;errors}^{protocol}$; otherwise, it is discarded. This process is depicted in Figure 6:

### 3.6. Receiver Message Handling and Report Generation

Once decoded in the receiver, messages are used to maintain an up-to-date list of detected drones and its associated positions. This list is then periodically reported to the UTM system with a frequency of $f_{report}^{protocol}\;\left[Hz\right]$. No further data processing is considered in our model. Posterior tracking and extrapolation are not included here.

## 4. Model Specification for Existing Surveillance Systems

In this section, the general model proposed in Section 3 is particularized for each of the surveillance systems/protocols described in the state-of-the-art review. The following table (Table 4) defines the numerical parameters as extracted from the technical specification or using typical values otherwise. Then, a series of subsections discuss these parameters and cover the remaining protocol-dependent model functions.

### 4.1. ADS-B

ADS-B does not use any MAC control. Within commercial ADS-B deployments for manned aviation, collisions are avoided using spatial diversity; however, in this case, receivers work in an isolated way. Thus, the probability of collision is that of an Aloha channel, as per [49]:

(14) $$P_{busy}=0;\;P_{collision\;known\;nodes}=0$$

(15) $$P_{collision\;hidden\;nodes}=P_{collision\;ALOHA}=1-e^{-2G_{S^{receiver}}}$$

Regarding the BER model, ADS-B uses PPM modulation. In [52], the relationship between the BER and the $\frac{E_{b}}{N_{0}}$ is defined as:

(16) $$\mathrm{BER}=\frac{1}{2}e^{-\frac{1}{2}\cdot \frac{E_{b}}{N_{0}}}\;$$

### 4.2. FLARM

The FLARM standard defines CSMA as the MAC protocol. A channel is sensed as being busy by a given emitter if any other emitter in $S^{emitter}\;$ transmits in the previous $T_{prop}+T_{pckt}$ seconds. This probability can be computed as (see [49] for a detailed analysis):

(17) $$P_{busy}=1-e^{-\left(1+a\right)G_{S^{emitter}}}$$

where *a* is the normalized propagation delay to the furthest node in each set:

(18) $$a=\frac{T_{prop}\;\left[s\right]}{T_{pckt}\;\left[s\right]};\;T_{prop}\;\left[s\right]=\frac{d_{\max\;}\left[m\right]}{c};\;c=3\times 10^{8}\frac{m}{s}$$

The collision probability in the receiver for CSMA-aware emitters can be obtained as:

(19) $$P_{collision\;known\;nodes}=1-\frac{e^{-aG_{S_{sensed}}}}{G_{S_{sensed}}\left(1+2a\right)+e^{-aG_{S_{sensed}}}}$$

Finally, the collision probability for the hidden nodes is that of the Aloha channel:

(20) $$P_{collision\;hidden\;nodes}=1-e^{-2G_{S_{hidden}}}$$

GFSK is used as modulation in FLARM protocol, as per [53]; the BER-$\frac{E_{b}}{N_{0}}$ relation is also:

(21) $$\mathrm{BER}=\frac{1}{2}e^{-\frac{1}{2}\cdot \frac{E_{b}}{N_{0}}}\;$$

### 4.3. RemoteID—Bluethoot Physical Interface

As is the case in ADS-B, no MAC procedure is used in Bluetooth broadcasting [38]. Therefore, the collision probabilities depicted in Section 4.1 are also applicable here. With respect to the BER, GFSK modulation is usually used. Therefore, the expression defined in Section 4.2 for FLARM is also used here.

### 4.4. RemoteID—WiFi Physical Interface

According to [39], CSMA is used in the WiFi subprotocol used by RemoteID. Thus, the expressions derived for FLARM in Section 4.2 are to be used here. For the BER computation, [54] proposes a simplified expression for the QPSK-OFDM modulation used in WiFi:

(22) $$\mathrm{BER}=\frac{1}{1+\frac{T_{p}}{T_{p}+T_{g}}\cdot \frac{E_{b}}{N_{0}}\;};\;T_{p}=0.0032;\;T_{g}=0.0008$$

## 5. Results

The collaborative surveillance models described in Section 4 have been implemented and integrated in a simulation tool for UTM systems proposed by authors in [12]. This consists of a distributed, agent-based modelling framework that allows one to replicate the input information (e.g., operation definition, telemetry reporting from drones, track reporting from surveillance networks, etc.) required by UTM systems for their operation. Thus, it allows for evaluating UTM systems without requiring real tests by simulating the behavior and interactions of relevant actors, such as pilots, GCSs, drones, surveillance networks and communication networks, that are individually modelled. The framework describes a model-agnostic, extensible architecture that allows to integrate multiple models for each actor and provides a set of tools to define simulation scenarios manually or randomly (i.e., list of flights, sensor’s locations…). Although simple simulation models were proposed in [12] for those actors, the work presented here for collaborative surveillance greatly extends these models, making them much more realistic.

Integrating the advanced models into the simulation tool allows one to use the proposed models in realistic, complex multi-drone scenarios, extending the accuracy of the original platform. Moreover, it is possible to assess and compare the performance of the different collaborative sensors we have considered. In particular, a base simulation scenario is proposed in this section where an area of interest is to be surveilled with different collaborative sensors. Thus, collaborative surveillance sensors are deployed within an airport where ATC controllers must ensure that nearby unmanned traffic does not interfere with manned traffic. The deployment consists of five different sensors (i.e., FLARM, ADS-B, RID with legacy BT PHY, RID with ER PHY, RID with WiFi PHY) located in the same position within the airport for comparison.

**Figure 7:**
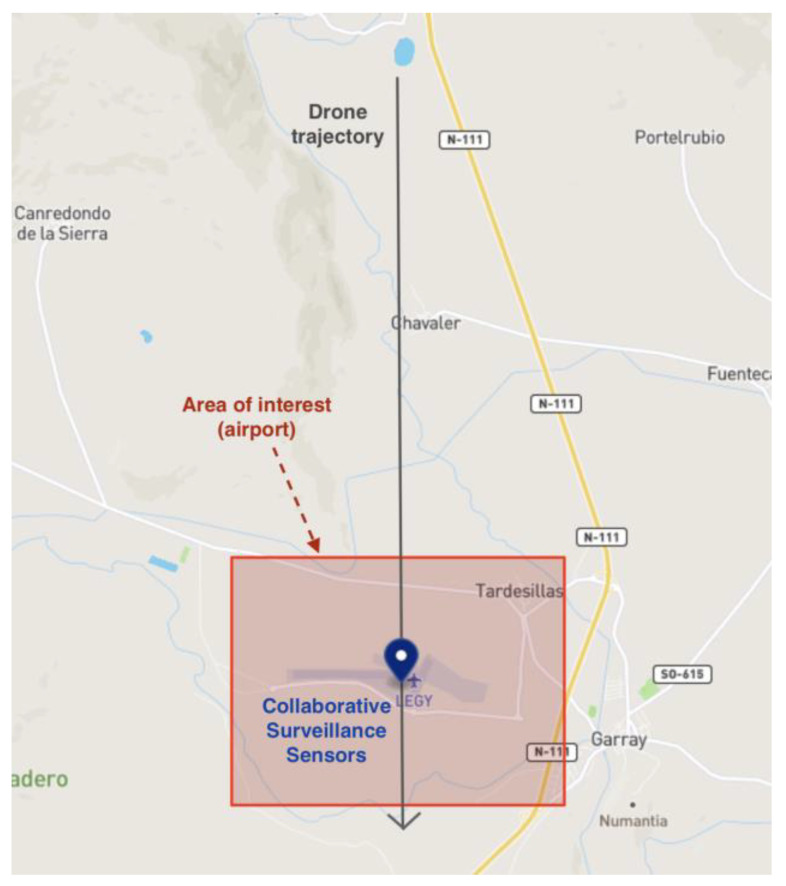
Geographical definition of the simulated scenario.

**Figure 8:**
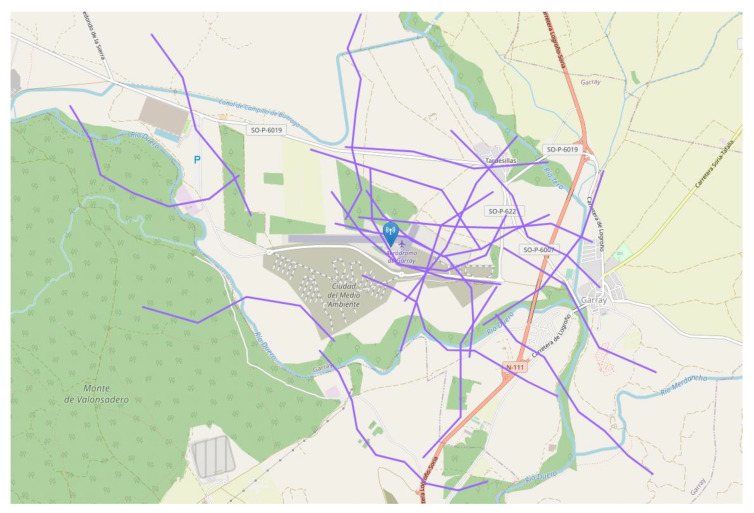
Twenty randomly generated flights for situation 2.

Over this base scenario, two different simulation situations are considered. The first one, depicted in Figure 7, aims to showcase the range, resolution, and periodicity related effects by simulating just one drone approaching the interest area. This drone is equipped with the corresponding emitter for each of the protocols so that it can be detected by all sensors. The second scenario consists of simulating an increasing number of drones in an area over time so that the spectrum-congestion effects can be observed. These flights are randomly generated, as depicted in Figure 8, within the area of interest with a set of utilities provided by the used simulation tools. The random generation of flights was performed using the utilities offered by the platform in [12], which generates random drone operations as a series of waypoints in a given area chosen by the user (other parameters such as the mean length of each flight or their time distribution can also be selected). In this case, drones are also outfitted with emitters for all protocols.

**Figure 9:**
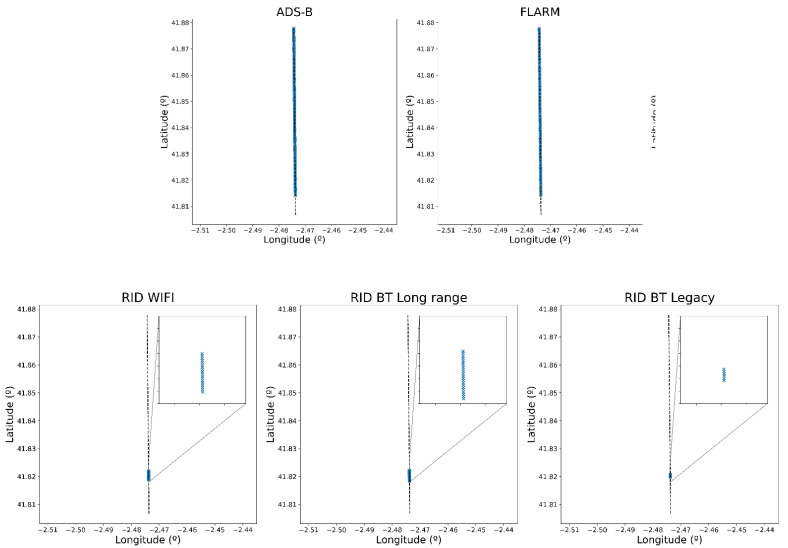
Plots returned by each sensor in situation 1. Drone trajectory is shown as a dashed line and plots are shown as blue crosses.

Both situations (i.e., drone trajectory, sensor location) were represented and executed in real time using the simulation platform. After running it, the plots generated by each of the sensors were retrieved for analysis. Starting with the first situation, Figure 9 shows the detections made by each sensor allowing to compare the different ranges of each protocol and package loss due to decoding errors. We can see that the detection limit is abrupt and almost no packet losses are observed, meaning that the effect of transmission errors due to low $\frac{E_{b}}{N_{0}}$ is negligible when also considering the receiver sensitivity and the FEC correction capacity. As expected, the RemoteID protocol’s range is lower than the ADS-B and FLARM ranges. Within RemoteID’s physical layers, WiFi and Bluetooth Long range achieve the best results.

**Figure 10:**
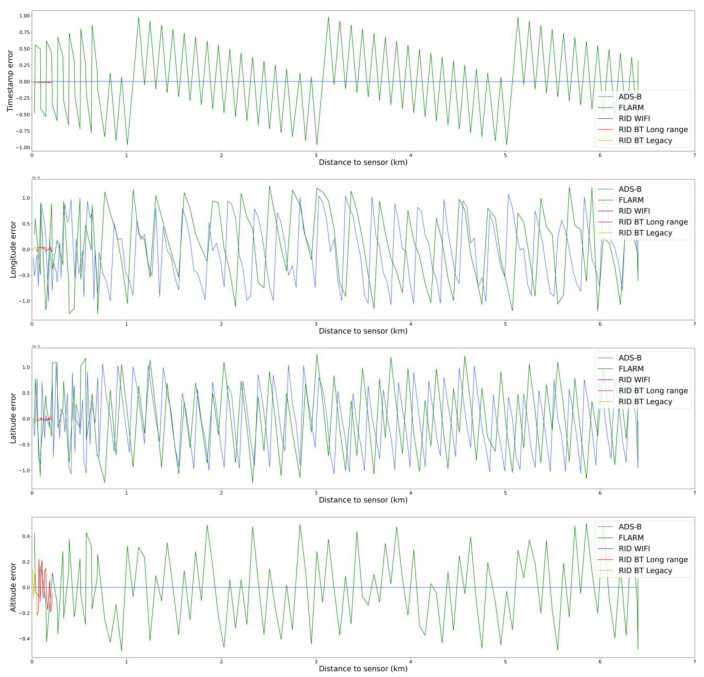
Errors due to encoding resolution for each sensor type.

Focusing on the positioning error, Figure 10 shows the difference between the GNSS position obtained by the drone and measurement received by each sensor. Contrary to what it is typical with non-collaborative sensors, positioning errors do not increase with distance to the sensor, as the position is not estimated by the sensor but forwarded by the drone. Errors are due to either encoding information with different resolutions or missing packages. The effect of the first type of error can be observed in Figure 10. The FLARM protocol encodes information with the worst resolution and thus achieves worse precision than the rest of sensors, particularly in the timestamp encoding. The shapes of the errors are typical of sensing systems dominated by quantification effects.

**Figure 11:**
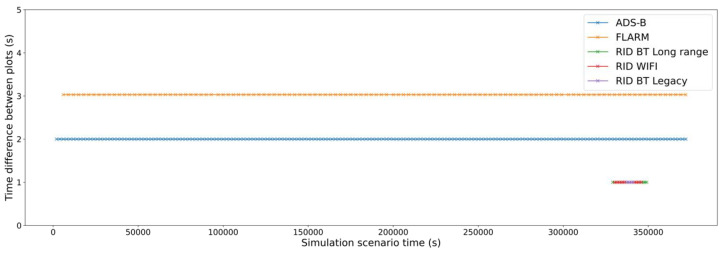
Evolution of plots periodicity (time difference between consecutive plots).

The second type of error corresponds to missing or delayed plots that decrease the receiving periodicity. This might be due to either decoding errors (caused by low Eb/N0) or network congestion (caused by either collisions or collisions avoidance). In this case, as only one drone is flown, collisions are not expected. This can be seen in Figure 11—the time between message arrivals is constant depending on the modelled message frequency of each protocol, and losses due to decoding errors are almost negligible for all the systems.

**Figure 12:**
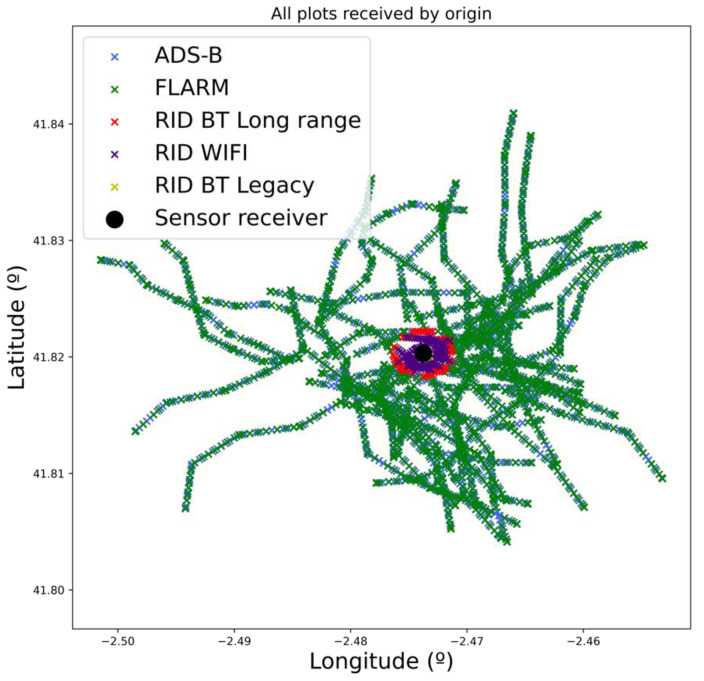
Plots received from all sensors and for all drones in situation 2.

Moving to the multidrone situation, the objective of this situation is to assess the expected congestion-related effects in a realistic scenario in terms of drone density. According to [55], the drone traffic over Paris is estimated to reach around 20,000 hourly flights by 2035. This yields an approximated drone density of 190 $\frac{\mathrm{flights}}{h\cdot {\mathrm{km}}^{2}}$. In our proposed scenario, the surveilled area, shown in Figure 8, covers 2.65 km^2^, and the scenario duration is limited to 5 min for practical reasons. Thus, applying the drone density derived from [55], around 42 flights would be expected within the area across the simulation. However, to encompass future drone usage growth and to clearly show the congestion trend for each technology, 50 drones were simulated. An example of all received plots is shown in Figure 12. Once again, the different effective ranges of each protocol can be observed. FLARM and ADS-B can cover greater areas, whereas RemoteID-based protocols only cover the proximity of the sensor. 

**Figure 13:**
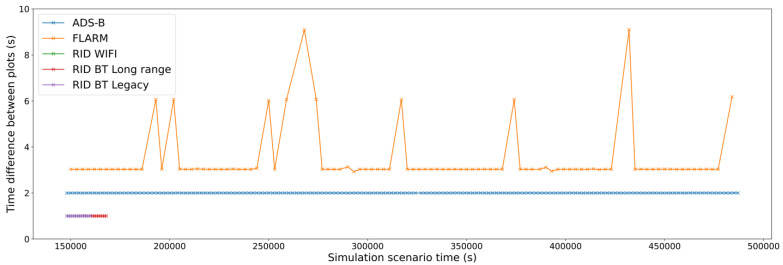
Periodicity of received messages from a given drone in a multidrone scenario (Situation 2).

If we focus on one of those drones, we can analyze how spectrum congestion due to the high number of drones affects the periodicity of its messages. In Figure 13, we can observe how the collision effect is relevant for the FLARM protocol with intervals between received messages of 9 s (which means that at least two consecutive packages have been discarded). It is also possible to see smaller time deviations due to retransmissions when the channel is sensed as being busy. 

**Figure 14:**
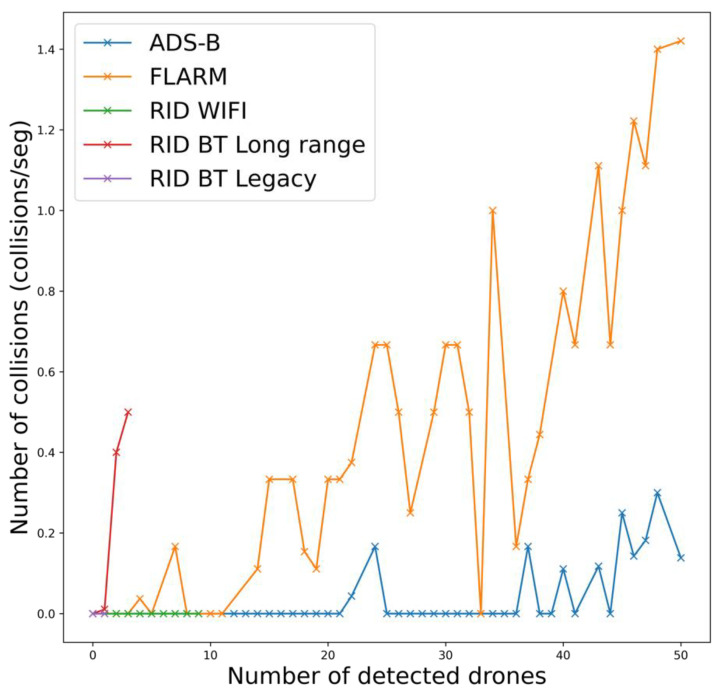
Collision rate for each sensor as the number of detected drones increases.

Finally, the overall effect of collisions for all drones can be assessed in Figure 14. There, the evolution of the number of collisions as the number of drones increases is plotted. In general, as the number of detected drones increases, so does the number of collisions. It might be expected that the performance of ADS-B would be worse than FLARM as it does not use a MAC protocol. However, channel capacity is far greater in the latter protocol yielding to a less occupied channel. The same effect can be observed by comparing RemoteID and Bluetooth’s physical layers: the long-range version achieves greater distances at the expense of a lower data rate yielding to higher channel occupancies for the same number of drones. The lower range of the RemoteID protocol is also prone to fewer collisions than FLARM or ADS-B, as for a given drone density it will detect fewer drones.

Summing up, the conducted experiments show that the expected effects contemplated in the proposed model are successfully simulated. In addition, we show that the most relevant effects are those related to the quantification and potential saturation of the channels leading to collisions and delays. Table 5 compares (with information from the obtained results and the protocols standards) the performance of each of the assessed technologies for different metrics: the size, weight and power requirements (SWaP), the achieved coverage range, the achieved information resolution, and the drone density each technology can handle. SWaP requirements (mainly power) are vital as drones are constrained platforms with limited energy and payload weight. ADS-B arises as a high-performance (both in resolution and coverage) alternative for high-density scenarios. However, its high SWaP requirement (mainly the high transmission power) may hinder its usage in many commercial drones. As an alternative, FLARM may be used for long-range surveillance at the expense of lower resolution for medium density scenarios. Finally, the purpose-built RemoteID surveillance technology is only suitable for low–medium-range surveillance. Particularly, the WiFi implementation of this protocol can work in high-density scenarios while maintaining a similar resolution as ADS-B and with a lower SWaP requirement. 

When comparing these protocols with direct telemetry reporting technologies via public mobile networks, it is expected that range will not be an issue in most non-rural scenarios due to the almost ubiquitous nature of those networks. Medium access-related problems are also not expected to be relevant thanks to the high capacity of modern mobile networks. Thus, the main effects will be those of different quantifications (although resolution will probably be higher) and a higher end-to-end latency.

## 6. Conclusions

Collaborative surveillance technologies have become a key technological enabler of UTM systems as they provide vital tactical information to monitor drone operations. Multiple technologies and protocols have already been proposed, and others are still under development to cover this technological gap. This paper has reviewed the technical specification and requirements of the most promising protocols including ADS-B, FLARM, Telemetry reporting and RemoteID. In addition, it has also discussed the main trends and existing discussing topics (i.e., identification number generation, networked vs. direct identification approach) guiding the development of these technologies. 

As a major information source to UTM systems, the availability and performance of cooperative surveillance greatly affect the performance of UTM systems. To assess this interaction, simulation models are a great tool to perform evaluations in a flexible and cost-effective way. Thus, this paper proposes a protocol-agnostic, statistical simulation model that considers effects such as measurement quantification, medium access congestion, signal propagation, transmission errors and signal decoding. This paper greatly extends our previous work in [12], where only the effect of the sensor range (using a simple pass-no pass model) was considered. Then, authors show how this model can be implemented for the reviewed surveillance protocols to simulate the detection process of each technology. This particularization for ADS-B, FLARM and RemoteID also surpasses the verisimilitude of the simple model proposed in [12], where the differentiating characteristics of each technology were not considered. 

Finally, the proposed models are integrated into a simulation platform previously proposed by the authors, enabling experimentation in realistic scenarios. In addition, this integration also benefits the preexisting platform as it enables testing and analyzing the integration of surveillance sensors into a UTM platform. Thus, it allows one to look for unexpected effects that can have a critical impact on security. Finally, it has been possible to carry out a comparative analysis of the different technologies and to demonstrate which are the main effects driving surveillance performance. Simulation results show that quantification and the potential saturation of the communication channels are the main effects. 

To our knowledge, there does not exist previous literature providing an overview on UAV-oriented collaborative surveillance networks. Likewise, the simulation of these networks, which will become key for UTM operational deployments, has not been addressed before. Thus, this paper includes several novel contributions that have not been previously covered in the existing literature:

- Technical review of existing collaborative surveillance technologies for UTM systems.
- Definition of a protocol-agnostic statistical simulation model for collaborative sensors for UTM.
- Parametrization of the proposed model for the different existing protocols.
- Integration of the proposed, advanced sensor models into a UTM evaluation platform.
- Experimentation within the said platform demonstrating that the most relevant effects are those related to quantification and potential saturation of the channels.

Overall, the work in this paper greatly extends our previous, preliminary work in the matter [12], providing a comprehensive, technology-agnostic, statistical model for collaborative sensors. In fact, it does not just offer a theoretical model, but it provides specific, practical implementations of it for the main existing technologies. Moreover, another key advantage of the proposed work is that it has been integrated within an existing UTM evaluation platform. This not only allows for evaluating collaborative sensors in realistic scenarios, but also to analyze how this information (or the lack of it) affects the performance of UTM systems. 

However, some limitations exist in the proposed model and should be addressed in future publications. These limitations are mainly related to the assumptions we made to provide a simple statistical model while encompassing all of the main effects. These limitations are:

- The propagation model. The free-space propagation model that has been considered may be valid for drones flying in open environments, but it is not valid for urban environments or areas with obstacles. Thus, advanced models (e.g., ray tracing) considering effects such as multipath, fading, etc., could be considered to improve the model.
- Interference modelling. Another drawback of the proposed model is that communication channels are considered to be independent and isolated from other RF sources. The effect of external interferences within the same band (i.e., other WiFi users, other emissions in ISM band…) should be considered to provide more realistic results. Likewise, bandwidth usage within protocols and the interaction between different surveillance protocols (i.e., RemoteID physical layers may concurrently use the same bandwidth) should also be better analyzed.
- Error correction. Finally, error correction modelling has been greatly simplified, not encompassing the enhanced correction capabilities of bitstream-based FEC.

It must be noted that while implementing enhanced models not suffering from the previous limitations would improve the accuracy of the model, it would also increase its computational requirements. As a result, this would probably hinder the ability to perform real time simulation, rendering the model useless for its integration with a UTM evaluation tool (that works in real time). Therefore, further work to address these points would need to contemplate a delicate tradeoff between a more accurate model and a practical one.

## Figures and Tables

**Table 1:** ADS-B main technical characteristics.

ADS-B Main Technical Characteristics		
General features	Communication type	Broadcast, message based
	Transmitter location	UAS
Physical layer	Transmission frequency (MHz)	1090 (978 preferred below 18.000 feet)
	Maximum transmission power (dBm)	A0: 48.5–51.5
		A1/A2: 51–54
		A3: 53–56
	Maximum physical bit rate (kbps)	1000
	Medium Access Protocol	None. The packets can interfere, alleviated deploying a large number of distributed receivers
	Modulation	Pulse position modulation (PPM)
Message codification	Message types	Short squitter (56 bits) for identification, long squitter (112 bits) for identification + data
	Information encoding resolution	High-integrity GNSS
	Message frequency	0.5 Hz (up to 2 Hz)
Other features	Authentication	No
	Encryption	No
	Error detection	Yes
	Error Correction	Yes

**Table 2:** FLARM UAS Electronic ID main technical characteristics.

FLARM UAS Electronic ID Main Technical Characteristics			
General features	Communication type	Broadcast, message based	
	Transmitter location	UAS	
Physical layer	Transmission frequency (MHz)	868.4	
	Maximum transmission power (dBm)	14	
	Maximum physical bit rate (kbps)	50	
	Medium Access Protocol	CSMA without collision detection (random initial wait of 0–1000 ms, random wait after transmission detection of 15–150 ms)	
	Modulation	2-Gaussian Frequency Shift Keying (GFSK)	
Message codification	Message types	UAV eID message, operator eID message	
	Message format and size (in bytes)	Preamble (4B) + Sync (3B) + Payload (24B) + CRC (2B) + Optional signature	
	Information encoding resolution	Timestamp	2 s
		Latitude	0.000025°
		Longitude	0.000025°
		Altitude	1 m (MSL or WGS84)
	Message frequency	0.33 Hz (or 1 Hz if position deviation is greater than 30 m)	
Other features	Authentication	Optional with external unspecified registration service	
	Encryption	Symmetric encryption (same key for all devices)	
	Error detection	Yes	
	Error correction	No	

**Table 3:** ASTM F3411-19 main technical characteristics.

ASTM F3411-19 Main Technical Characteristics				
General features	Communication type	Broadcast, message based		
	Transmitter location	UAS		
Physical layer	Physical protocol	Legacy BT	BT 5.x long range	WiFi
	Physical frame/message supporting RemoteID payload	Beacon broadcast message	Extended advertisements (with FEC for x4 range)	Service discovery frame based on neighbor awareness networking
	Physical frame overhead [B]	23	35	21
		(ASTM standard [35] defines how the RemoteID payload is to be inserted within each physical frame. The protocol overhead understood as the frame length minus the payload is depicted here.)		
	Transmission frequency (MHz)	2400 (channels 37, 38, 39)	2400 (all BT channels)	2400 (ch. 6) 5800 (ch. 149)
	Maximum transmission power (dBm)	+5	+5	+11 (2400 MHz)+4 (5800 MHz)
	Medium Access Protocol	No medium access protocol [38].		Non-persistent CSMA [39]
	Modulation	Gaussian Frequency Shift Keying (GFSK) (other modulation schemes are considered in BT specification such as differential phase shift keying that allow increased data rates not needed for this application.)		Orthogonal frequency-division multiplexing (OFDM) with QAM symbol mapping. (Multiple modulation schemes are considered in compliance with the different IEEE 802.11 protocols, OFDM-based protocols are the most widespread nowadays)
Message codification	Message types (only compulsory)	Basic ID message (UAS identification) Location message (UAS position)		
	Message format and size (in bytes)	Header (1B) + Message data (24B) [payload of aforementioned frames]		
	Information encoding resolution	Timestamp		0.1 s
		Latitude		1.0 × 10^−6^°
		Longitude		1.0 × 10^−6^°
		Altitude		0.5 m
		Speed		0.25 m/s if speed < 0.5 knts.0.75 m/s otherwise
	Message frequency	Basic ID message: at least 0.33 HzLocation message: at least 1 Hz		
Other features	Authentication	Optional with external unspecified registration service		
	Encryption	No		
	Error detection	Physical layer dependent (Yes)		
	Error correction	No	Yes (convolutional FEC)	Yes

**Table 4:** Model parameters for each surveillance system.

Model Parameter	ADS-B	FLARM	RemoteID-BT Legacy	RemoteID-BT ER	RemoteID-WiFi
P	{timestamp, longitude, latitude, altitude, speed}	{timestamp, longitude, latitude, altitude}	{timestamp, longitude, latitude, altitude, speed}		
$r_{timestamp}\;\left[s\right]$	0.0078125	2	0.1		
$r_{longitude}\;\left[{}^{{}^{\circ}}\right]$	0.0000215	0.000025	1.0 × 10^−6^		
$r_{latitude}\;\left[{}^{{}^{\circ}}\right]$	0.0000215	0.000025	1.0 × 10^−6^		
$r_{altitude}\;\left[m\right]$	0.0047625	1	0.5		
$r_{speed}$ [*m*/*s*]	0.2315	N/A	0.75		
$f_{position}\;\left[Hz\right]$	0.5	0.33	1		
$f_{identification}\;\left[Hz\right]$	N/A	N/A	0.33		
$L_{payload}^{protocol}\;\left[B\right]$	14	24	25		
$L_{overhead}^{protocol}\;\left[B\right]$	0	9	23	35	21
$T_{max\;age}^{protocol}\;\left[s\right]$	N/A	1	1		
$T_{backoff}\;\left[s\right]$	N/A	0.15	N/A	N/A	0.01
$R^{protocol}\;\left[bps\right]$	1.0 × 10^6^	50,000	1.0 × 10^6^	1.25 × 10^5^	2.4 × 10^7^
FEC	1/6	1	1	1/4	1/2
$P_{tx}\;\left[dBm\right]$	48.5	14	5	5	11
$f_{tx}\;\left[GHz\right]$	1.09	0.8684	2.4	2.4	2.4
$S_{rx}\;\left[dBm\right]$	−72	−95	−70	−82	−85
$S_{tx}\;\left[dBm\right]$	−72	N/A	N/A	N/A	−85
$F_{eq}\;\left[dB\right]$	10	10	10	10	10
$N_{max\;errors}$	7	0	0	7	7
$f_{report}\;\left[Hz\right]$	1	1	1	1	1

**Table 5:** Comparison of collaborative surveillance technologies with three different performance levels for each metric: high, medium and low.

Performance Metric	ADS-B	FLARM	RemoteID-BT Legacy	RemoteID-BT ER	RemoteID-WiFi
SWaP requirements	High	Medium	Low	Low	Low
Coverage Range	High	High	Low	Medium	Medium
Resolution	High	Low	High	High	High
Drone density	High	Medium	Low	Low	High

## Data Availability

Not applicable.
